# Domestic Dogs in Rural Communities around Protected Areas: Conservation Problem or Conflict Solution?

**DOI:** 10.1371/journal.pone.0086152

**Published:** 2014-01-20

**Authors:** Maximiliano A. Sepúlveda, Randall S. Singer, Eduardo Silva-Rodríguez, Paulina Stowhas, Katharine Pelican

**Affiliations:** 1 College of Veterinary Medicine, University of Minnesota, St. Paul, Minnesota, United States of America; 2 Instituto de Medicina Preventiva Veterinaria, Facultad de Ciencias Veterinarias, Universidad Austral de Chile, Valdivia, Chile; 3 Departamento de Ecología y Biodiversidad, Facultad de Ecología y Recursos Naturales, Universidad Andrés Bello, Santiago, Chile; 4 Facultad de Ciencias Veterinarias y Pecuarias, Universidad Mayor, Santiago, Chile; University of Illinois at Urbana-Champaign, United States of America

## Abstract

Although domestic dogs play many important roles in rural households, they can also be an important threat to the conservation of wild vertebrates due to predation, competition and transmission of infectious diseases. An increasing number of studies have addressed the impact of dogs on wildlife but have tended to ignore the motivations and attitudes of the humans who keep these dogs and how the function of dogs might influence dog-wildlife interactions. To determine whether the function of domestic dogs in rural communities influences their interactions with wildlife, we conducted surveys in rural areas surrounding protected lands in the Valdivian Temperate Forests of Chile. Sixty percent of farm animal owners reported the use of dogs as one of the primary means of protecting livestock from predators. The probability of dog–wild carnivore interactions was significantly associated with the raising of poultry. In contrast, dog–wild prey interactions were not associated with livestock presence but had a significant association with poor quality diet as observed in previous studies. Dog owners reported that they actively encouraged the dogs to chase off predators, accounting for 25–75% of the dog–wild carnivore interactions observed, depending on the predator species. Humans controlled the dog population by killing pups and unwanted individuals resulting in few additions to the dog population through breeding; the importation of predominantly male dogs from urban areas resulted in a sex ratios highly dominated by males. These results indicate that dog interactions with wildlife are related to the role of the dog in the household and are directly influenced by their owners. To avoid conflict with local communities in conservation areas, it is important to develop strategies for managing dogs that balance conservation needs with the roles that dogs play in these rural households.

## Introduction

Dogs were domesticated over 14,000 years ago [1], and as human populations have grown, so have populations of dogs. Currently, the world dog population is estimated at over 700 million [2]. In developing countries, free-ranging dogs in rural areas are of particular concern as human populations expand into borderland and natural areas in search of affordable and productive farm land [3], [4], [5]. Recent studies have found that rural dogs, particularly free-ranging dogs, can threaten wildlife through predation, competition or as sources of infectious diseases [2], [3], [5], [6]. At the same time, dogs are being promoted as a nonlethal strategy for abating human-wildlife conflict in some areas [7], [8], [9], [10], [11]. For example, guardian dogs can protect livestock from carnivore predation, thus reducing human-wildlife conflict and protecting vulnerable carnivore populations from retaliatory killing [8]. While these studies typically have focused on the interaction between domestic dogs and wildlife or the efficacy of using guardian dogs against wild predators, to date little research has been conducted regarding the reasons rural families keep dogs and how these roles impact dog-wildlife interactions. A specific understanding of the motivation for dog ownership in rural communities and how dog populations are managed is needed to reduce the likelihood of detrimental or unpopular dog management policies and strategies around conservation areas.

Our objective was to test whether the role and management of dogs influences the nature and frequency of dog-wildlife interactions. Using a survey of households in rural communities in Chile, we investigated the relationship between farm management practices and farmer-reported dog-wildlife interactions. In addition, the survey provided important information on dog management and demography, both relevant for improving dog population management in the future, particularly in wildlife conservation areas. By examining the role of dogs from a subsistence farming perspective, this study provides key insights into the role of the dog in farming and conservation. In so doing it elucidates the critical but socially sensitive tradeoff between dog as farm-protector and dog as wildlife threat.

## Methods

### Ethics Statement

Oral informed consent was provided by an adult in each interviewed household in a standardized format. The general purpose of the survey was explained in Spanish to each research participant. Consent was obtained by having the interviewee state that they agreed to participate. The data was analyzed anonymously. The use of this survey and approach was approved by the Institutional Review Board at the University of Minnesota (1304E31141).

### Study Area

**Figure 1:**
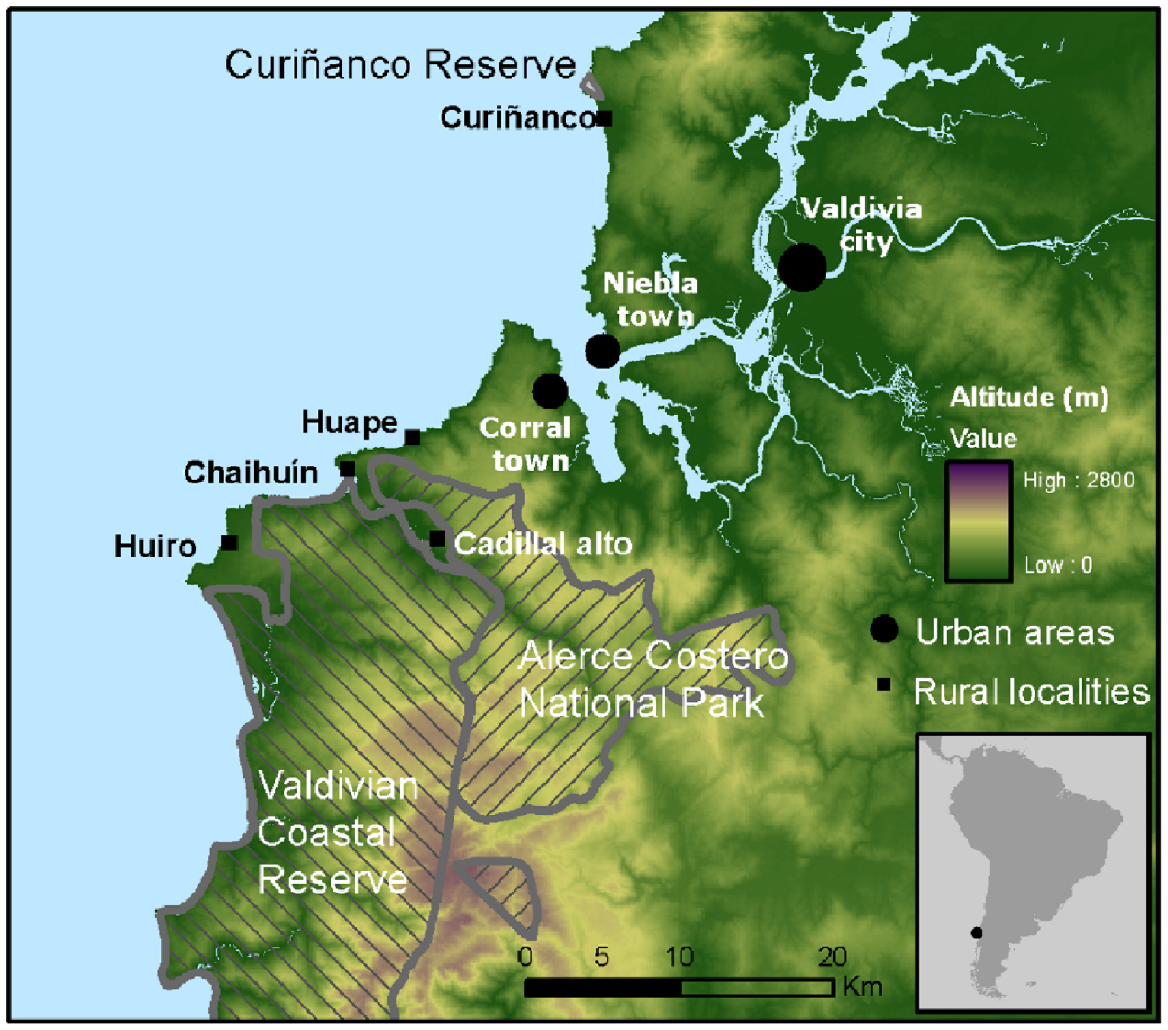
Map of the study area in the Coastal Range of southern Chile.

The study area was located in the coastal range of the Chilean Temperate Forest between latitude 39°41′ S and 39°58′ S. Between 11 and 76 households were interviewed in 5 rural communities (Curiñanco, Huape, Chaihuin, Huiro and Cadillal Alto) adjacent to three different protected areas (Curiñanco Reserve, Alerce Costero National Park and the Valdivian Coastal Reserve [VCR]) (see Fig. 1). The economy of these rural communities is based mainly on extraction of marine resources (seafood, algae), livestock production (primarily cattle, sheep and poultry) and forest resource utilization (timber and native berries). Poverty levels are high in all five rural communities [12], [13]. Land cover in these areas is primarily characterized as native evergreen forest located within protected areas in the higher elevations, grazing pastures in the lowlands and outside of the protected areas, and exotic plantations (eucalyptus and pine) mostly outside of the protected areas (except in the VCR where eucalyptus plantations are also present). Average annual rainfall is 2.5 m per year and annual average temperature is 12°C (range: 8°–17°C) [14]. All of the localities were within the Valdivian Ecoregion, an area that has been labeled a global biodiversity priority [15].

Free-ranging dogs are common in the study area, and both predation on the Southern pudu (*Pudu puda*) and European hare (*Lepus europaeus*) and interference competition with the chilla fox (*Lycalopex griseus*) have been reported [16], [17]. Other wild mammal species (>1 kg) present in the study site and potentially affected by the presence of dogs are the guigna (*Leopardus guigna*), puma (*Puma concolor*), Molina’s hog-nosed skunk (*Conepatus chinga*), lesser grison (*Galictis cuja*), southern river otter (*Lontra provocax*), marine otter (*Lontra felina*), coypu (*Myocastor coypus*), invasive American mink (*Neovison vison*) [12], [13] and Darwin’s fox (*Lycalopex fulvipes*) [18]. Species of conservation concern are the southern river otter (endangered), marine otter (endangered), guigna (vulnerable), Darwin’s fox (critically endangered) and the southern pudu (vulnerable) [19].

### Survey Implementation

From October to December 2010 we attempted to interview one person per household in the five rural communities (Fig. 1). The total number of households estimated by local park rangers was 245. In instances where we did not find anyone at home during a scheduled visit, we conducted up to two additional visits. Information collected included the number of dogs per household, dog management practices including the type of food provided and whether there was any restraint or control of dog movement of the dogs (i.e. completely free-roaming or restricted to some degree), dog demographics (age, sex, reproduction, mortality and source of dogs), presence of farm animals (poultry, cattle, sheep), observed dog attacks/harassment of wildlife, and measures for farm animal protection, including dogs. The questionnaire included 15 questions and was based on surveys used for studies with similar objectives [16], [20] (see Questionnaire S1 in Supplementary Material). We did not expect that farmers would avoid questions related to dog-wildlife interactions since previous work in the region found that residents openly reported wildlife interactions including killing threatened species [21].

### Dog Utility and Wildlife Interactions

We used the survey to determine whether dogs were used by their owners to protect poultry and sheep against wild predators. We quantified the wildlife species and domestic dog/wildlife interactions each respondent observed during the prior year. A dog-wildlife interaction was defined as killing or harassment by the dog of: a) carnivores (including mink, fox, guigna and puma), referred to as dog-carnivore interaction, or b) herbivores or prey animals, particularly European hare and Southern pudus, referred to as dog-prey interaction. To assess the potential influence of the owner in the dog-carnivore interaction, we determined the percentage of dog owners who reported chasing off a particular predator species using dogs.

### Statistical Analysis

We used generalized linear models with a logit link function to assess the probability of dog-carnivore and dog-prey interactions [22]. A binary response variable was based on the dog owner reporting the presence/absence of a dog interaction with wildlife during the last year. Two different outcomes were modeled; one for dog-wild carnivore interactions (dog-carnivore, hereafter) and another one for dog- prey interactions. For both models, we used the following predictor variables: 1) number of dogs per house (DOGS), 2) chicken ownership (POULTRY); 3) sheep ownership (SHEEP); 4) locality (SITE), and 5) whether the dogs were fed commercial dog food (FOOD). The variable SITE was restructured into a binary variable based on whether the household was located within the Curiñanco locality.

We hypothesized that a higher number of dogs (DOGS) would increase the probability of dog-wildlife interactions at the household level. Site was used as a covariate because of differing proximity to urban areas at each locality (*i.e.* habitat composition around localities). Curiñanco (SITE) was used as the reference site because this locality was closest to the main city of Valdivia (Fig. 1) and therefore possessed less forest coverage and more urban influence that could potentially explain differences in human behaviors. Chickens (POULTRY) are preyed upon by most small wild carnivores in the study area (guigna, American mink, lesser grison and foxes), while sheep (SHEEP) are mostly preyed upon by the puma. Other farm animals such as cattle are infrequently attacked by predators [21]. Finally, the consistent use of commercial dog food (FOOD) was included because previous studies have shown that poorly fed dogs kill wild vertebrates more often than well-fed dogs [16]. We assumed that the consistent use of commercial dog food was a better diet than alternatives such as wheat bran or leftovers-based diets.

To determine significant predictors for dog-carnivore and dog-prey interactions we used an information-theoretic approach. Models were created with all possible combinations of the predictor variables. For model selection purposes we utilized the Akaike information criterion corrected for small sample size (AICc). We calculated Akaike differences (ΔAICc) and weights (*wi*) to determine the relative support for a model given the data for the set of candidate models. To account for model selection uncertainty we averaged the estimates of the coefficients of the main effect variables across all candidate models. To determine the relative importance of the predictor variables we calculated the sum of the Akaike weights over all of the candidate models in which the parameter of interest appeared [23]. The magnitude of the effect of each predictor variable on the response variable was determined by the odds ratio, defined as the ratio of the odds of an outcome in a group to the odds of the outcome in a second group [22], [24], and 95% confidence interval of averaged estimates. Goodness of fit for the global candidate models and best selected model for dog-carnivore and dog-prey interactions was assessed using the unweighted sum of squares test [25]. All analyses were performed in R [26] (R code provided in Supplementary Material).

## Results

We interviewed 146 households (approximately 59.6% of estimated households); only two people did not agree to participate. Of respondents, 123 had dogs and provided information about dog demography and management (Table 1). We observed an adult-dominated dog age structure, highly skewed towards males, with a total population size of 218 among the interviewed households. During the year 2010 only 14 dogs (6.4% of total dogs) were added to the population through breeding (recruitment) out of the 39 pups born. Some of the dog owners indicated that females in heat are problematic because they attract male dogs, resulting in dog fights and negatively affecting the body condition of these working dogs.

**Table 1:** Demography and management of rural dogs.

*Demographic parameters*	*Responses*
Number of households interviewed	146
Households with dog ownership (%)	123 (84.2)
Total dogs	218
Average number of dogs per household (SE)	1.7 (0.06)
Average dog age in years (SE)	4.5 (0.02)
Male: female ratio	7.4∶1
Number of litters in prior year	9
Number of total pups	39
Pup survival rate before 2 months old	0.36^a^
Number of dogs recruited from breeding	14
Percentage of male dogs castrated	1.6
Percentage of female dogs neutered	26.6
Percentage of dogs obtained locally	44.9
Number of imported dogs less than 1 year old	27^b^
Number of adult dog mortalities	53^c^
*Household management*	
Percentage of dogs allowed to roam free	92.2
Percentage of households that used commercialdog food	52.0
Percentage of dogs used for farm animal protection	69.7

^a^ 36% of the pups that died were killed by the dog owner;

^b^ 92.6% males and 7.4% females;

^c^ 7.5% killed by the dog owner.

Demography and management of domestic dogs in rural areas around three protected areas in Southern Chile.

In this study, we refer to dogs brought from other localities as ‘imported’ dogs in order to differentiate them from stray dogs that are adopted by the household. Importation of dogs (*n* = 27) from nearby towns or localities was a more important input to the population than recruitment (12.4% versus 6.4%), and most of these imported dogs were males (Table 1). Dog owners reported killing unwanted pups from litters as well as adult dogs that attacked domestic animals (Table 1). A significantly higher proportion of respondents were willing to spay female dogs compared to neutering male dogs (63.6% vs. 17.1%; *P*≤0.005), and more female dogs were neutered than males (Table 1). In the previous year, 53 adult dogs died due to the following causes: illness (30.2%), motor vehicle collisions (26.4%), old age (20.8%), killed by owner (7.5%) and from unknown reasons (15.1%). In addition, 3 dogs disappeared. The proportion of people who reported having killed a dog at least once in their lifetime was 27.2, all related to attacking or killing farm animals. Nine households reported killing 14 different dogs (not owned by them) during the previous year because they had killed farm animals.

No feral dogs (dogs completely independent of human resources [3]) were reported by the respondents or observed by the research teams throughout the study period. However, most dogs in the survey were allowed to roam free (92.2%). Dog diet was based primarily on homemade food (73.2%), commercial food (52.0%) and wheat bran (40.75%). When asked about food provision for the dogs during the previous day, 6.5% of dog owners reported that no food had been provided. Less than half the respondents vaccinated (33.6%) or dewormed (41.9%) their dogs.

**Figure 2:**
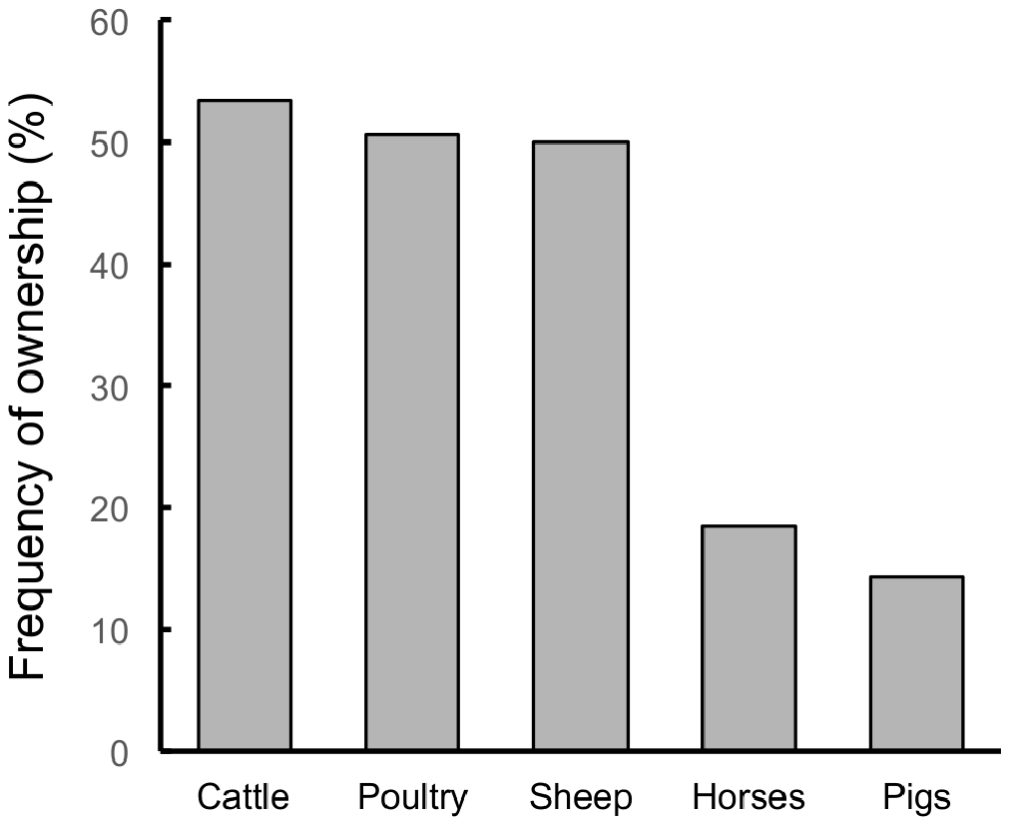
Farm animal ownership. Farm animal ownership in rural households around three protected areas in southern Chile.

**Figure 3:**
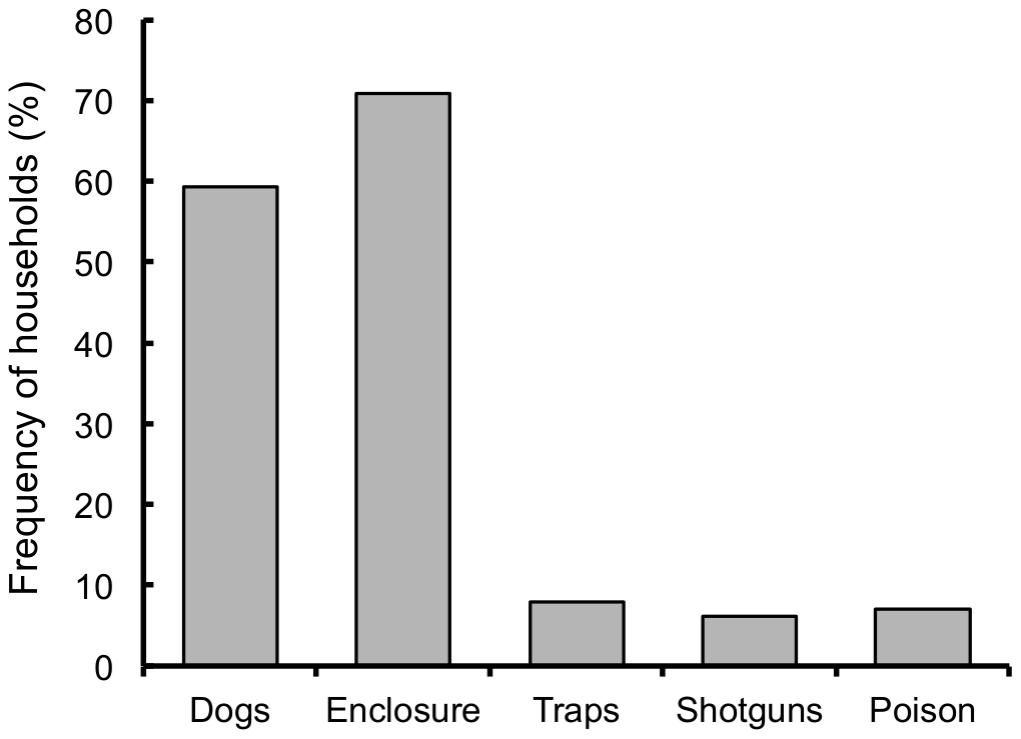
Measures of farm animal protection in rural areas in southern Chile. Measures of farm animal protection against carnivore predators reported by rural interviewees around three protected areas in southern Chile.

Seventy-seven percent of interviewed households owned at least one kind of farm animal. Poultry, cattle and sheep were the most common farm animals (Fig. 2), and the most common means of protecting these animals against predators were enclosures and use of dogs (Fig. 3).

Respondents reported having seen most of the listed wildlife species during the previous year (but note that we asked about foxes in general). Exceptions include lesser grison and coypu that were observed by only two and nine respondents, respectively. Dog-wildlife interactions during the previous year were reported for all species (Table 2) with the exception of the grison and river otter where no interactions were reported. Most interactions reported were detected near the household, although the precise distances were not recorded. During the study period, dog owners were observed rescuing two Southern pudus from dog attacks and bringing them to the VCR facilities for veterinary support. During informal conversations with four different farmers, it was consistently expressed that the farmers do not like dogs that grow accustomed to making long-distance hunting forays because they believe that this behavior negatively affects the dog’s body condition, and if repeated frequently will reduce the dog’s utility in guarding the household and the farm animals.

**Table 2:** Dog-wildlife interactions reported by dog owners.

Species	Number households with dog interactions (%)
*Poultry predators*	
Fox	22 (17.9)
Guigna	10 (8.1)
American mink	6 (4.9)
Lesser grison	0
*Livestock predator*	
Puma	13 (10.6)
*Other carnivores*	
River otter	0
Marine otter	1 (0.8)
Molina’s skunk	8 (6.5)
*Herbivores*	
European hare	73 (59.3)
Southern pudu	6 (4.9)
Coypu	1 (0.8)

Number of households with dogs (n = 123) indicating dog-wildlife interactions observed during the previous year.

**Figure 4:**
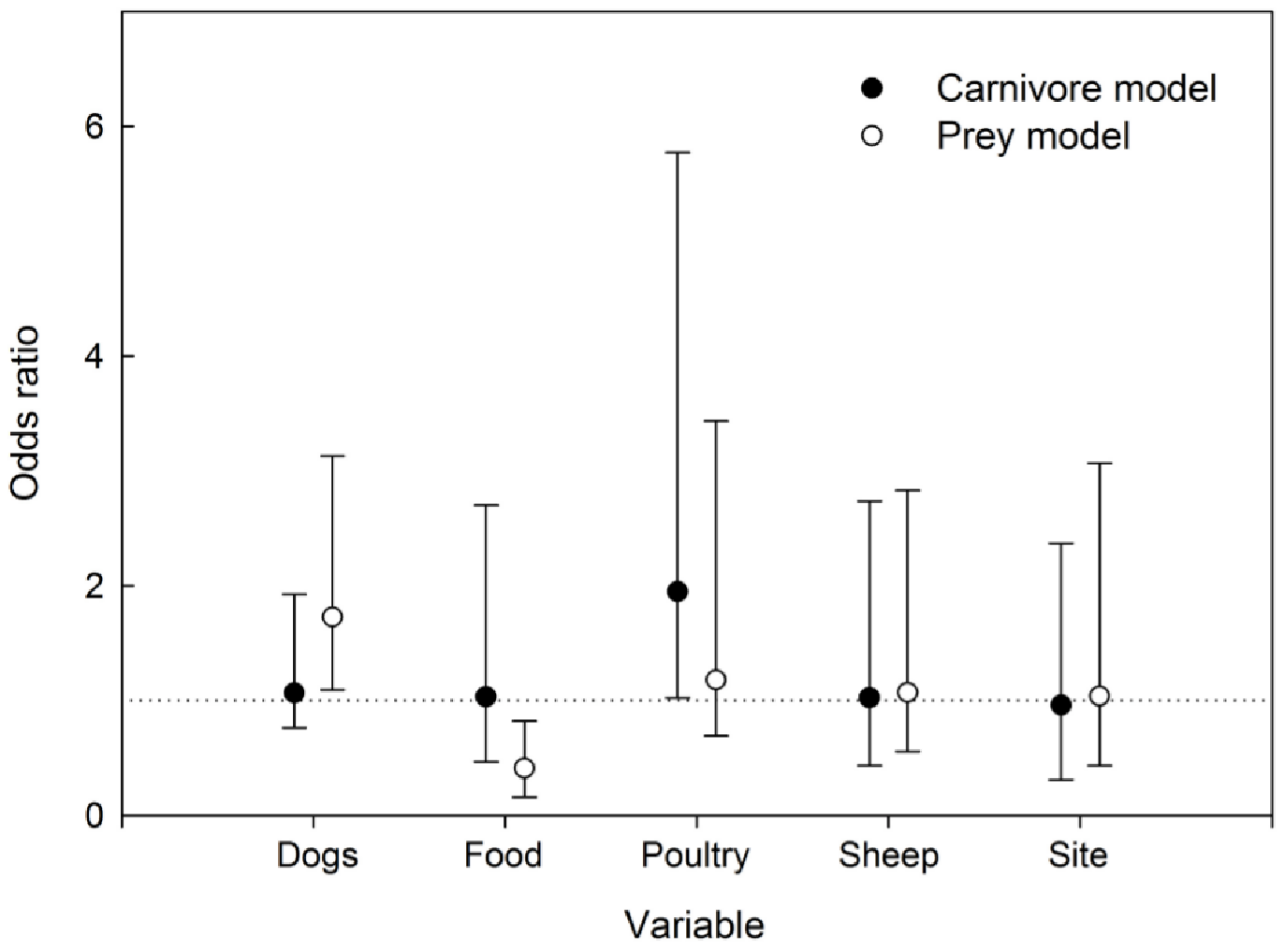
Model-averaged odds ratios of rural dogs interacting with carnivore and prey species. Model-averaged parameter estimates shown as odds ratios and 95% confidence intervals for explaining rural dog interactions with carnivore and prey species. Odds Ratios <1 indicate a negative association with occurrence while Odds Ratios >1 indicate a positive association with occurrence.

The variables included in the best-ranked models (ΔAICc <2) to explain the farmer-reported dog-carnivore interactions included POULTRY and DOGS. Averaged parameter estimate of presence of poultry in the household was positively associated with dog harassment and killing of carnivores (see Table S1 for all competing models). A marginally positive association with the probability of a dog-carnivore interaction was also attributable to the number of dogs (DOGS; Fig. 4). Adequate food (FOOD), locality (SITE) and sheep ownership (SHEEP) were poor predictors for dog harassment of carnivores (Fig. 4). For the dog-prey interactions, the best candidate models (ΔAICc <2) included all of the predictors. Averaged parameter estimates for dog-prey interactions were positively associated with DOGS and negatively associated with FOOD (Fig. 4 and Table 3).

**Table 3:** Best models to estimate the probability of owned dog interactions with carnivores and prey species.

	Competing models	*K*	AICc	ΔAICc	ωi
Dog-carnivore interactions	POULTRY	2	144.45	0.00	0.21
	DOGS+POULTRY	3	146.03	1.58	0.09
					
Dog-prey interactions	DOGS+FOOD	3	158.25	0.00	0.27
	DOGS+POULTRY+ FOOD	4	159.25	1.00	0.16
	POULTRY+DOGS+SHEEP	4	159.92	1.67	0.12
	DOGS+SITE+FOOD	4	160.24	1.99	0.10

Summary of model selection to estimate the probability of owned dog interactions with carnivores and prey species. Models are ranked by AICc values. Columns include the number of variables (K), Akaike’s Information Criterion (AICc), distance from the lowest AICc (Δ AICc), and Akaike’s model weight (ωi). Models showed include only those with a ΔAICc ≤2. See table1 in Supplementary material for all competing models.

Among farm animal owners, the percentages of dog-carnivore interactions that were motivated by dog owners actively encouraging the dog to chase off a predator species were 75%, 62.5%, 38.9% and 25% for American mink, puma, fox and guigna, respectively. The only dog-prey interaction that was motivated by humans was the European hare, where 10% of the total interactions were encouraged by dog owners.

## Discussion

This study investigated dog – wildlife interactions in the context of farm management and dog utility. In this study rural dogs were predominantly owned and free-roaming and their interactions with wildlife were significantly influenced by their role on the farm as well as by their management. Dogs appeared to interact with carnivores at the encouragement of their owners in the context of protecting livestock. In contrast, dog interactions with prey species were mostly undesirable from the farmer’s perspective and seemed to be driven more by hunger and inadequate diet. The skewed dog sex ratio and high rate of externally-acquired dogs showed that the dog population was heavily managed by farmers, indicating a potential opportunity for informed interventions aimed at minimizing interactions with wildlife.

Davlin and VonVille [27] found that rural areas in developing countries have more houses with dogs and more free-roaming dogs than urban areas, a situation consistent with the current study. In this study, people strongly selected males over females thereby generating a strong bias in terms of sex ratios and, as a consequence, a low reproductive rate and poor recruitment of new animals to the population through breeding. This skewed sex ratio is consistent with other studies of domestic dogs in developing countries with increasing male bias in rural areas such as Sri Lanka [28], Mexico [29], Madagascar [30] and Chile [16], [31]. The skewed sex ratio in the present study was influenced by culling as well as the acquisition of predominantly male dogs from other areas into this population, female dogs being considered difficult to manage and detrimental during periods of estrus to the work capacity of the males. A third, but minor means of control in this population was surgical sterilization. When used, it was targeted at females, possibly to minimize management problems associated with estrus. Even though, in general, there are marked behavioral differences between male and female dogs [32], there is some evidence that working dog performance is not affected by the sex of the dog [33]. In support of this, farmers informally stated motivation to avoid female dogs was associated with their reproductive behavior (estrus and pregnancy), and not their performance. Given the management of the dogs and the low number of females in this study area, neutering programs would likely have little effect on the already human-controlled dog population but might improve dog welfare to address dog killings by humans (unwanted bitches) if accompanied by educational programs.

In our study we found that the farmer-reported interactions between dogs and wild animals were associated with their management and role on the farm. Interestingly, the models that best explained dog attacks on carnivores included poultry and the number of dogs, whereas the models that best explained interactions with prey included type of food provided and the number of dogs as the strongest predictors. Having a greater number of dogs seemed to be associated with a higher frequency of dog attacks on wild animals. This may be related to sampling effects (i.e., the probability of observing at least one dog attacking wildlife is higher for owners of multiple dogs) or to pack hunting behaviors. Silva-Rodriguez and Sieving [16] reported that well fed dogs killed prey species less often than those fed on low-quality food such as wheat bran or household leftovers. While our study detected a similar association for dog-prey species interactions, we also showed that dog-carnivore interactions were better predicted by livestock ownership than food provision.

The fact that dog-prey and dog-carnivore interactions were predicted by different variables has important implications for wildlife conservation. Minimizing impacts on prey will require different dog management strategies compared to impacts on carnivores in communities surrounding wildlife sanctuaries. In terms of dog-carnivore interactions, dogs that belonged to households with livestock had a higher likelihood of interacting with carnivores. In these circumstances, the use of dog breeds selected specifically for certain behavioral traits (i.e. shepherd or guardian breed dogs) could potentially minimize wildlife-human conflict and retaliatory killing of wildlife. Domestic dogs have been used to guard livestock against predator attacks throughout recorded human history [7] and continue to be used in this manner worldwide [7], [8], [9]. In a study by González et al. [8] that was conducted in the northern Patagonian steppe of Argentina, 37 puppies of mixed-breed guarding dogs were delivered to 25 herders. These dogs were effective at reducing both goat losses and retaliatory killing. In the current study, dogs were used to protect poultry and other livestock, and dog-carnivore interactions were directly related to this key function on the farm. In fact, dog owners encouraged their dogs to chase carnivores seen as threats to their livestock. The fact that the presence of dogs is perceived by farmers to reduce carnivore-caused livestock losses may also result in a reduced retaliatory killing of carnivores, suggesting that dogs whose primary function is to guard livestock indirectly contribute to reducing human-caused mortality of carnivores [8].

The situation with dog-carnivore interactions is in contrast to the situation with dog-prey interactions as identified in this study. Here, dog-prey interactions are correlated with food quality and, for the most part, are perceived by the dog owner as negative and distracting the dog from the primary function of livestock and homestead protector. These behaviors are clearly detrimental to wildlife populations. For example, a study in New Zealand documented a single German shepherd that was estimated to have killed approximately 500 kiwis (*Apteryx australis*) out of a population of 900 within a six-week period [34]. Recent work by Silva-Rodríguez and Sieving [35] using camera traps in the same study area as our current study determined that dogs shaped the distribution of pudu by lethal (predation) and non-lethal (avoidance) means. In this study, dogs occasionally harassed or killed pudus despite the fact that people held highly positive attitudes toward pudus [21] and actively tried to save pudus from dog attacks. In addition, some interviewees reported that their dogs killed domestic livestock, which is a frequent problem in southern Chile [36] and elsewhere [37], [38, [39], further expanding the potential unintended negative consequences of domestic carnivores. Thus, although dogs can play an important role in protecting livestock and farms from wildlife pests [7], [7], [9], [10], [11], and thereby minimize human-wildlife conflict and retaliatory killing of carnivores [8], dogs also have direct impacts on wildlife and livestock populations through predation [2], [5]. This hunting activity is generally considered undesirable by dog owners and is an activity that can be minimized through a change in diet and management. This provides an interesting opportunity to manage dog populations in a way that maximizes wildlife conservation while also protecting the rural farmers’ way of life and livelihoods.

One limitation of our study is the reliance on human accounts of interactions between dogs and wildlife rather than observing these interactions directly. The data obtained in this study will be skewed by what people see and are willing to report. However, during a study conducted simultaneously in the same area we found that dogs were mostly diurnal and spent most of their time around houses [40]. These findings are in line with results reported by Woodroffe & Donnelly (2012) [41] in Africa, where dogs can be seen by their owners during the majority of their “active time.” Second, most interactions reported correspond to dog attacks on hares and (likely chilla) foxes. Both species are rarely seen in forested habitat in the area and prefer more open habitats (those habitats that occur near human households). Most interactions would be expected to occur near houses, thereby increasing the likelihood that they will be detected by the dog owners. Finally, the data that are being analyzed reflect events observed at least once during the previous year. Considering that hares are common near houses, it would be unlikely for a dog used to hunt to have no reported interactions with prey in the previous year.

The fact that most dogs added to this rural population were obtained from localities outside of the study areas also is important information for future management interventions. Because dogs are imported anyway, interventions could focus on improving the health and behavior of these imported animals. Interventions could include the provision of well selected dog breeds allowing a potential increase in the effectiveness of the guarding behavior while minimizing the problems caused by these dogs [7], [42]. Imported dogs could also be vaccinated for diseases of concern to wildlife prior to importation to minimize risk of disease transfer to endangered wildlife. The adequate training of dogs is one of the key aspects in rearing livestock-protection dogs [7], [8], [9]. This, and other aspects (neutering, vaccination, adequate feeding, etc.) should be emphasized by gobernmental and/or non-governmental conservation agencies in education campaigns. Given the effort that is being undertaken to import dogs into these relatively remote locations, access to new, high quality, vaccinated dogs could lead to even greater uptake by the community.

Clearly, as dog populations grow, it will be critical to improve dog management close to sensitive conservation areas. At the same time, dogs clearly play a critical role as working members of households (e.g., herding, guarding, and hunting) in rural communities (Table 1) [7], [43]. Thus, improving management of problematic behaviors in rural dogs around protected or natural areas will require a balanced approach that integrates the significant benefit that dogs provide for rural residents into the management strategy. By engaging rural communities directly, this study shows that dog roles and management in the household are significant factors in dog-wildlife conflict. By understanding these drivers of interaction, interventions can be targeted to optimize beneficial behaviors for the farmer while minimizing detrimental wildlife interactions. This provides an important model for future management of anthropogenic pressure on conservation areas.

## Supporting Information

Table S1 **Summary of model selection to estimate the probability of owned dog interactions with carnivores and prey species.** Models are ranked by AICc values. Columns include the number of variables (*K*), Akaike’s Information Criterion (AICc), distance from the lowest AICc (Δ AICc), and Akaike’s model weight (ωi).
(DOCX)

Questionnaire S1 **Dog’s owner questionnaire.**
(DOCX)
