# Supplementary material for: Domestic Dogs in Rural Communities around Protected Areas: Conservation Problem or Conflict Solution?
Source: PLoS One. 2014 Jan 20;9(1):e86152. doi: 10.1371/journal.pone.0086152 (PMC3896434; doi:10.1371/journal.pone.0086152)
Supplement: Table S1 — Summary of model selection to estimate the probability of owned dog interactions with carnivores and prey species. Models are ranked by AICc values. Columns include the number of variables (K), Akaike’s Information Criterion (AICc), distance from the lowest AICc (Δ AICc), and Akaike’s model weight (ωi). (DOCX) [file pone.0086152.s001.docx]

**Table S1.** Summary of model selection to estimate the probability of owned dog interactions with carnivores and prey species. Models are ranked by AICc values. Columns include the number of variables (*K*), Akaike’s Information Criterion (AICc), distance from the lowest AICc (Δ AICc), and Akaike’s model weight (ωi).

|  | Competing models | *K* | AICc | ∆AICc | ωi |
| --- | --- | --- | --- | --- | --- |
| Dog-carnivore interactions | POULTRY | 2 | 144.45 | 0.00 | 0.21 |
|  | DOGS+POULTRY | 3 | 146.03 | 1.58 | 0.09 |
|  | POULTRY+SITE | 3 | 146.46 | 2.01 | 0.08 |
|  | POULTRY+FOOD | 3 | 146.48 | 2.03 | 0.08 |
|  | POULTRY+SHEEP | 3 | 146.55 | 2.10 | 0.07 |
|  | INTERCEPT | 1 | 147.06 | 2.61 | 0.06 |
|  | DOGS | 2 | 147.94 | 3.49 | 0.04 |
|  | DOGS+POULTRY+SITE | 4 | 148.03 | 3.58 | 0.03 |
|  | DOGS+POULTRY+ FOOD | 4 | 148.08 | 3.63 | 0.03 |
|  | POULTRY+DOGS+SHEEP | 4 | 148.16 | 3.71 | 0.03 |
|  | SHEEP | 2 | 148.45 | 4.00 | 0.03 |
|  | POULTRY+SITE+ FOOD | 4 | 148.48 | 4.02 | 0.03 |
|  | POULTRY+SITE+SHEEP | 4 | 148.60 | 4.14 | 0.03 |
|  | POULTRY+ FOOD +SHEEP | 4 | 148.61 | 4.16 | 0.03 |
|  | FOOD | 2 | 149.12 | 4.67 | 0.02 |
|  | SITE | 2 | 149.12 | 4.67 | 0.02 |
|  | DOGS+SHEEP | 3 | 149.60 | 5.14 | 0.02 |
|  | DOGS+SITE | 3 | 150.01 | 5.55 | 0.01 |
|  | DOGS+ FOOD | 3 | 150.03 | 5.58 | 0.01 |
|  | DOGS+POULTRY+SITE+SHEEP | 5 | 150.21 | 5.75 | 0.01 |
|  | DOGS+POULTRY+ FOOD +SHEEP | 5 | 150.25 | 5.80 | 0.01 |
|  | SITE+SHEEP | 3 | 150.48 | 6.03 | 0.01 |
|  | FOOD +SHEEP | 3 | 150.54 | 6.09 | 0.01 |
|  | POULTRY+SITE+ FOOD +SHEEP | 5 | 150.65 | 6.20 | 0.01 |
|  | SITE+ FOOD | 3 | 151.21 | 6.76 | 0.01 |
|  | DOGS+SITE+SHEEP | 4 | 151.62 | 7.16 | 0.01 |
|  | DOGS+ FOOD +SHEEP | 4 | 151.72 | 7.26 | 0.01 |
|  | DOGS+SITE+ FOOD | 4 | 152.12 | 7.67 | 0.00 |
|  | DOGS+POULTRY+SITE+ FOOD +SHEEP | 6 | 152.28 | 7.83 | 0.00 |
|  | SITE+ FOOD +SHEEP | 4 | 152.59 | 8.14 | 0.00 |
|  | DOGS+SITE+ FOOD +SHEEP | 5 | 153.76 | 9.30 | 0.00 |
|  |  |  |  |  |  |
| Dog-prey interactions | DOGS+ FOOD | 3 | 158.25 | 0.00 | 0.27 |
|  | DOGS+POULTRY+ FOOD | 4 | 159.25 | 1.00 | 0.16 |
|  | POULTRY+DOGS+SHEEP | 4 | 159.92 | 1.67 | 0.12 |
|  | DOGS+SITE+ FOOD | 4 | 160.24 | 1.99 | 0.10 |
|  | DOGS+POULTRY+ FOOD +SHEEP | 5 | 161.33 | 3.08 | 0.06 |
|  | DOGS+SITE+ FOOD +SHEEP | 5 | 162.03 | 3.78 | 0.04 |
|  | DOGS | 2 | 162.44 | 4.19 | 0.03 |
|  | POULTRY+ FOOD | 3 | 162.88 | 4.63 | 0.03 |
|  | FOOD | 2 | 163.02 | 4.77 | 0.02 |
|  | DOGS+POULTRY | 3 | 163.03 | 4.77 | 0.02 |
|  | DOGS+POULTRY+SITE+ FOOD +SHEEP | 6 | 163.50 | 5.25 | 0.02 |
|  | DOGS+SHEEP | 3 | 164.03 | 5.78 | 0.02 |
|  | FOOD +SHEEP | 3 | 164.03 | 5.78 | 0.02 |
|  | DOGS+SITE | 3 | 164.55 | 6.29 | 0.01 |
|  | SITE+ FOOD | 3 | 164.76 | 6.51 | 0.01 |
|  | POULTRY+ FOOD +SHEEP | 4 | 164.78 | 6.53 | 0.01 |
|  | POULTRY+SITE+ FOOD | 4 | 164.84 | 6.59 | 0.01 |
|  | POULTRY+DOGS+SHEEP | 4 | 165.10 | 6.84 | 0.01 |
|  | DOGS+POULTRY+SITE | 4 | 165.15 | 6.90 | 0.01 |
|  | SITE+ FOOD +SHEEP | 4 | 166.02 | 7.77 | 0.01 |
|  | DOGS+SITE+SHEEP | 4 | 166.14 | 7.89 | 0.01 |
|  | POULTRY | 2 | 166.74 | 8.49 | 0.00 |
|  | POULTRY+SITE+ FOOD +SHEEP | 5 | 166.84 | 8.59 | 0.00 |
|  | DOGS+POULTRY+SITE+SHEEP | 5 | 167.24 | 8.99 | 0.00 |
|  | INTERCEPT | 1 | 167.43 | 9.18 | 0.00 |
|  | SHEEP | 2 | 168.36 | 10.11 | 0.00 |
|  | POULTRY+SHEEP | 3 | 168.64 | 10.39 | 0.00 |
|  | POULTRY+SITE | 3 | 168.84 | 10.59 | 0.00 |
|  | SITE | 2 | 169.47 | 11.22 | 0.00 |
|  | SITE+SHEEP | 3 | 170.45 | 12.20 | 0.00 |
|  | POULTRY+SITE+SHEEP | 4 | 170.77 | 12.52 | 0.00 |
