# Supplementary material for: Domestic Dogs in Rural Communities around Protected Areas: Conservation Problem or Conflict Solution?
Source: PLoS One. 2014 Jan 20;9(1):e86152. doi: 10.1371/journal.pone.0086152 (PMC3896434; doi:10.1371/journal.pone.0086152)
Supplement: Questionnaire S1 — Dog’s owner questionnaire. (DOCX) [file pone.0086152.s002.docx]

### SUPPLEMENTARY MATERIAL

#### **Questionnaire S1.** Dog’s owner questionnaire.

1. ¿Cuantos perros tiene en este momento? (hembras, machos). *(At present, how many dogs do you have? males and females).*
2. ¿Qué edad tienen?. (*How old are they?).*
3. ¿Cual es el origen de sus perros?. (*Where did you get them?).*
4. ¿Como maneja a sus perros? permanentemente libre, amarrado, libre solo de día. (*How do you manage your dogs? Continuously free, leashed, leashed only during the day*).
5. ¿Están vacunados o desparasitados?. (*Are they vaccinated or treated against parasites?*).
6. Durante el último año ¿cuantos perros ha tenido?. (*During the last year, how many dogs/cats have you had?*).
7. Durante el año pasado, ¿cuantos perros se han muerto? edad y causa. (*During the last year, how many of your dogs have died?. Age and cause of death*).
8. ¿A matado alguna vez un perro? ¿Fecha de la ultima vez? ¿Cual fue el motivo? (*Have you ever killed a dog? Date to the last time? What was the reason?*).
9. ¿Cuantas veces han parido las hembras en el último año? ¿Cuando?. (*During the last year, how many of the females gave birth? When?*).
10. ¿Cuantos cachorros en cada parto? ¿qué hizo con ellos?. (*How many puppies were born per litter? what did you do with them?*).
11. ¿Cual es el principal alimento de sus perros? ¿Cual fue su comida ayer? (*What is the main source of food of your dogs?* *What was their meal yesterday?)*
12. ¿Ha visto alguna vez alguno de estos animales? (Se mostraron 2 fotos para identificacion de cada especie silvestre) ¿donde? ¿cuando? ¿que hacían? ¿qué hizo usted?. (*Have you ever seen one of these named species? [Two pictures for each wild species were provided for identification] Where? When? What did they do? What did you do?*).
13. ¿Ha cazado o atacado su perro alguna de las siguientes especies en el último año? cuantos, donde, y cuando: Pudu, zorro, guiña, vison, chingue, huillin, coipo, liebre. (*During the last year, has your dog killed or attacked any of the following species? how many, where and when: Pudu deer, foxes, lesser grisson, mink, guigna, skunk, river otter, marine otter, coypu.*).
14. ¿Cuantos de los siguientes animales tiene (gallinas, ovejas, vacas, cerdos, cabras, otros)?. (*How many of the following animals do you have: hens, sheep, cows, pigs, goats, others*.)
15. ¿Qué medidas de protección toma contra predadores de sus animales domésticos? Armas, perros, veneno, encierro, trampas, otras, nada. (*What measures do you use to protect your farm animals against predators?: shotguns, dogs, poison, closure, traps, others, nothing.*)

*Interviewed person:*

*Sex: Female Male*

*Age (years):*

*Occupation:*

*Locality:*

*Number of people habiting the house: Adults ___ Children ___*
